# Association between global sagittal malalignment and increasing hip joint contact force, analyzed by a novel musculoskeletal modeling system

**DOI:** 10.1371/journal.pone.0259049

**Published:** 2021-10-28

**Authors:** Takanori Miura, Naohisa Miyakoshi, Kimio Saito, Hiroaki Kijima, Jumpei Iida, Kazutoshi Hatakeyama, Kotaro Suzuki, Akira Komatsu, Takehiro Iwami, Tosiki Matsunaga, Yoichi Shimada

**Affiliations:** 1 Department of Orthopedic Surgery, Kakunodate General Hospital, Akita, Japan; 2 Department of Orthopedic Surgery, Akita University Graduate School of Medicine, Akita, Japan; 3 Department of Rehabilitation Medicine, Akita University Hospital, Akita, Japan; 4 Department of Orthopedic Surgery, Omagari Kousei Medical Center, Akita, Japan; 5 Department of Mechanical Engineering, Akita University Faculty of Engineering Science, Akita, Japan; 6 National Institute of Technology (KOSEN), Sendai College, Sendai-shi, Miyagi, Japan; University of Memphis, UNITED STATES

## Abstract

Patients with adult spinal deformity have various standing postures. Although several studies have reported a relationship between sagittal alignment and exacerbation of hip osteoarthritis, information is limited regarding how spinopelvic sagittal alignment changes affect hip joint loading. This study aimed to investigate the relationship between sagittal spinopelvic-lower limb alignment and the hip joint contact force (HCF) using a novel musculoskeletal model. We enrolled 20 women (78.3±6.7 years) from a single institution. Standing lateral radiographs were acquired to measure thoracic kyphosis, lumbar lordosis, the pelvic tilt, sacral slope, sagittal vertical axis (SVA), femur obliquity angle, and knee flexion angle. In the model simulation, the Anybody Modeling System was used, which alters muscle pathways using magnetic resonance imaging data. Each patient’s alignment was entered into the model; the HCF and hip moment in the standing posture were calculated using inverse dynamics analysis. The relationship between the HCF and each parameter was examined using Spearman’s correlation coefficient (r). The patients were divided into low SVA and high SVA groups, with a cutoff value of 50 mm for the SVA. The HCF was 168.2±60.1 N (%BW) and positively correlated with the SVA (r = 0.6343, p<0.01) and femur obliquity angle (r = 0.4670, p = 0.03). The HCF were 122.2 and 214.1 N (75.2% difference) in the low SVA and high SVA groups, respectively (p<0.01). The flexion moment was also increased in the high SVA group compared with that in the low SVA group (p = 0.03). The SVA and femur obliquity angle are factors related to the HCF, suggesting an association between adult spinal deformity and the exacerbation of hip osteoarthritis. Future studies will need to assess the relationship between the hip joint load and sagittal spinopelvic parameters in dynamic conditions.

## Introduction

The spine and hip joint are anatomically and functionally adjacent via the pelvis, and changes in one joint can potentially clinically affect the other. The concept of the spine-hip relationship has defined the interaction between the lumbopelvic complex and the hip joint. Offierski and MacNab discussed the relationship between hip osteoarthritis (HOA) and lumbar spine disease and described the hip-spine syndrome (HSS). They categorized this syndrome into four groups: simple, complex, secondary, and misdiagnosed. In particular, the secondary group describes the interrelationship of spine symptoms and deformity of the hip [1]. With respect to secondary HSS, HSS is defined as a pathological condition wherein the primary pathological structure is the spine that affects the hip joint, and the sagittal spinopelvic alignment assessment is important to understanding hip-spine relationships [2, 3]. Patients with adult spinal deformity (ASD) have various standing postures with compensatory mechanisms, such as pelvic retroversion, hip extension, and knee flexion, to maintain the sagittal balance [4, 5]. Several studies have reported some relationship between sagittal spinal alignment and exacerbation of HOA, such as lumbar kyphosis and posterior pelvic tilt are more frequent in elderly onset HOA [6], and the larger anterior inclination of the spine in the standing position is associated with radiographic progression of HOA [7].

The decrease in the femoral head coverage area due to posterior pelvic tilt, impingement, and an increased hip joint load due to sagittal alignment changes have been reported as the reasons for the progression of HOA [7–11]. Hip joint contact force (HCF) is involved in the progression of HOA [12], and there has been a report on increased HCF due to posterior pelvic tilt according to the finite element method [13]. However, information is limited regarding how spinopelvic sagittal alignment changes affect hip joint loading. To clarify the relationship between changes in the sagittal alignment and hip joint load, biomechanical analysis is necessary.

HCF is measured *in vivo* with radio telemetry devices in the implanted prosthesis [14, 15]. However, the expense of measuring HCF *in vivo* and the need for subjects to undergo total hip arthroplasty at the same time limit the number of subjects that can be analyzed [16]. Therefore, the musculoskeletal model (MSM), which is capable of noninvasive HCF prediction, is a useful alternative to instrumented implants [17]. In previous research, various software packages, such as OpenSim and Anybody Modeling System (AMS), have been used [17, 18]. These models can be used to calculate the joint moments required to perform a given kinematic task using inverse dynamics calculations. Redundancy in the MSM can be resolved by using optimization algorithms to determine the optimal combination of muscle activity and force that produces the required moments [19, 20]. It has been reported that MSMs were used for estimating patient-specific HCF for various physical activities [16, 21, 22] and improving the accuracy of HCF prediction [20, 23]. However, to our knowledge, there have been no studies on the actual measurement of HCF in patients with ASD. Furthermore, although there have been reports of lumbar load changes with spinopelvic sagittal alignment [19], no studies have investigated the association between sagittal alignment and HCF using an MSM.

In the original AMS, the lumbar vertebrae are movable. However, the thoracic vertebrae and ribs are constructed as a rigid body [24]. Therefore, it was impossible to construct a patient-specific MSM in patients with ASD. Furthermore, in the estimation of HCF, accuracy was problematic because of inaccurate muscle attachment positions and non-anatomical muscle pathways [20, 25]; therefore, this study aimed to construct a patient-specific adapted MSM of the sagittal spinopelvic lower limb alignment in elderly women’s posture during standing and investigate the relationship between sagittal spinopelvic alignment and HCF.

## Materials and methods

### Patient-specific data

We enrolled 20 women (78.3±6.7 years) who visited a single institution for osteoporosis treatment between 2018 and 2020. The inclusion criteria were ambulatory patients who had been diagnosed with primary osteoporosis and had no experience of heavy work. The patients with a history of spine surgery, two or more vertebral fractures, hip or knee arthroplasty, osteoarthritis of the hip, and complaints of severe back or hip pain were excluded. The study was approved by the Institutional Review Board of Kakunodate General Hospital (approval number: 000612), and all patients provided written informed consent.

### Image acquisition and anatomical parameters extraction

**Fig 1:**
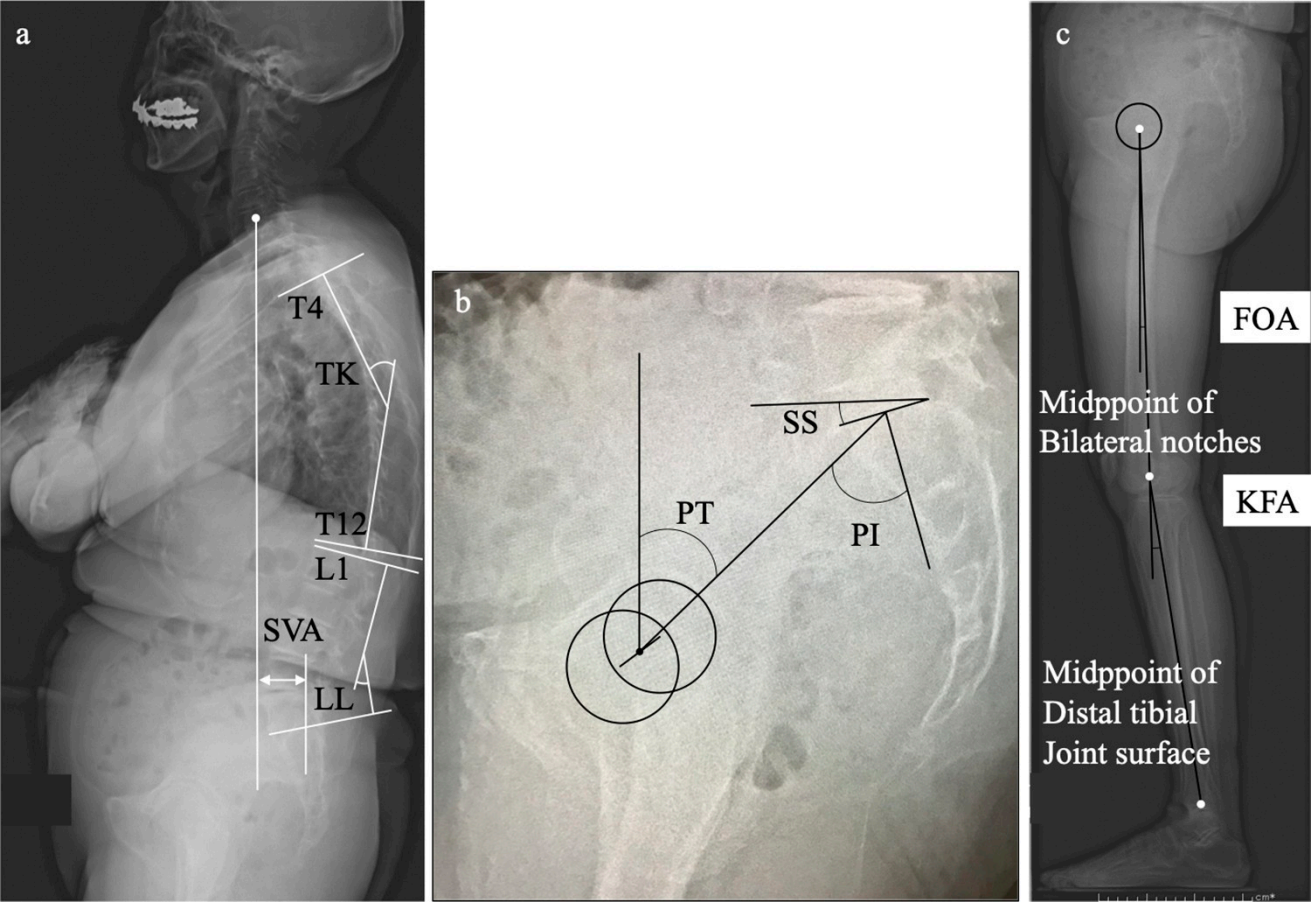
Sagittal alignment parameters in the standing position. Spinal parameters, TK (thoracic kyphosis), LL (lumbar lordosis), and the SVA (sagittal vertical axis), were measured on a lateral whole-spine radiograph (a). Pelvic parameters, the PT (pelvic tilt), SS (sacral slope), and PI (pelvic incidence), were measured (b). Lower limb parameters, the FOA (femur obliquity angle) and KFA (knee flexion angle), were measured (c).

Lateral radiographs of the whole spine and lower limb were taken with both hands placed on the clavicle in a relaxed standing position. Spinopelvic lower-limb alignment parameters were measured by a single author. Spinal parameters included in this analysis were thoracic kyphosis (TK: Cobb angle from the upper endplate of T4 to the lower endplate of T12), lumbar lordosis (LL: Cobb angle from the upper endplate of L1 to the lower endplate of S1), and the sagittal vertical axis (SVA: horizontal distance from the C7 plumb line originating at the middle of the C7 vertebral body to the posterior superior endplate of S1). Pelvic parameters included in this analysis were the pelvic tilt (PT: the angle between the line connecting the midpoint of the sacral plate to the bi-coxo-femoral axis and the vertical plane), sacral slope (SS: the angle between the sacral plate and the horizontal plane), and pelvic incidence (PI: the angle between the line perpendicular to the sacral plate and the line connecting the midpoint of the sacral plate to the bi-coxo-femoral axis). Lower limb parameters included in this analysis were the femur obliquity angle (FOA: the angle between the femoral shaft and the vertical line) [26] and knee flexion angle (KFA: the angle between the line from the hip axis to the midpoint of the bilateral notches of the femoral condyles and the line from the notch to the midpoint of the distal tibial joint surfaces) [27] (Fig 1).

The radiological criteria of ASD according to the International Spine Study Group include a frontal Cobb angle >20°, SVA >50 mm, TK >60°, and/or PT >25°. In the Scoliosis Research Society-Schwab ASD classification, the sagittal modifiers SVA, PT, and PI-LL are defined. The cut-off value of the SVA is 40 mm, and pelvic retroversion is defined as >20° of PT [28]. The knee flexion angle in the standing position in healthy subjects was <6° [27], and that >6° was defined as knee joint flexion.

### Musculoskeletal model

**Fig 2:**
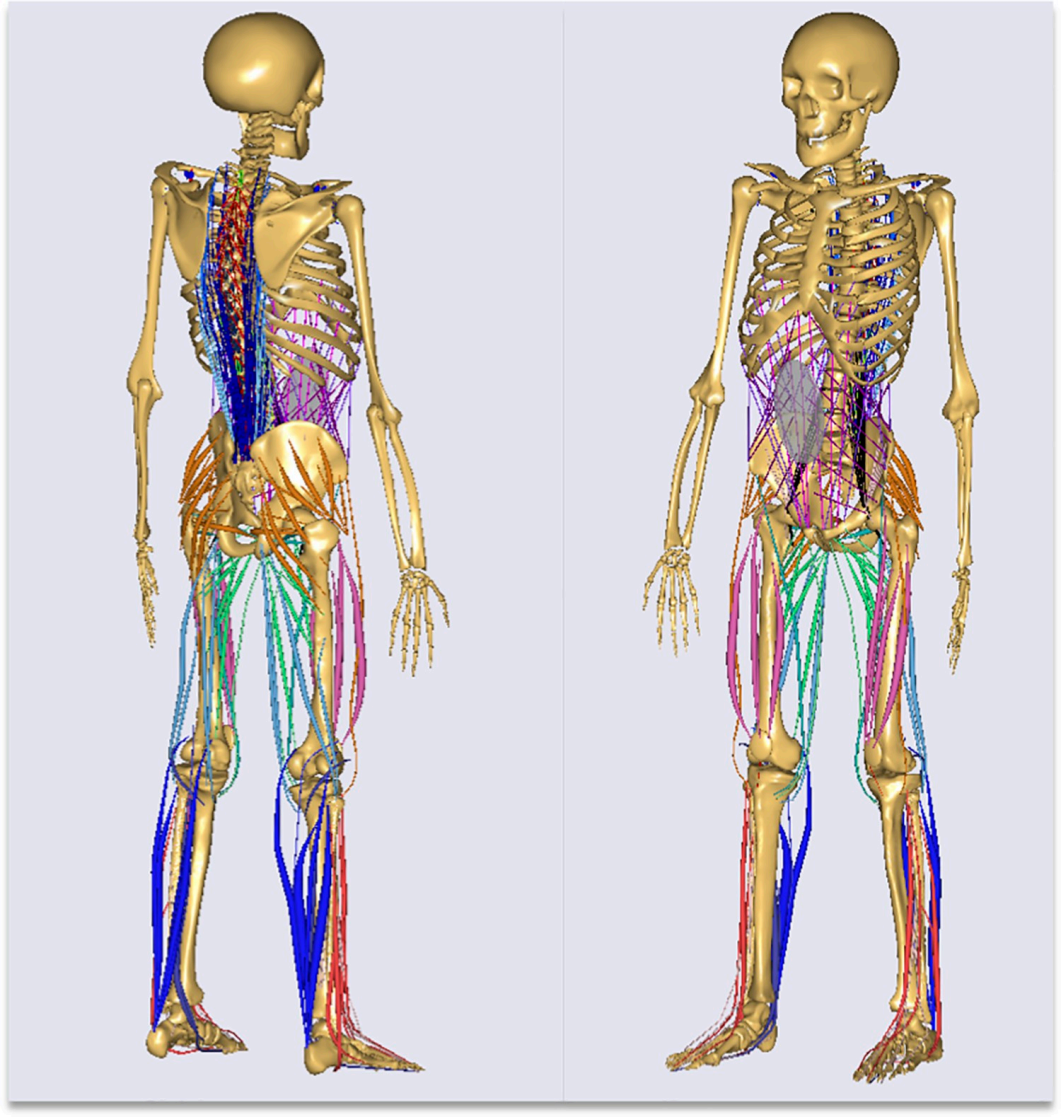
The constructed musculoskeletal model using the Anybody Modeling System. The constructed model altered the thorax, muscle attachment points, and pathways.

The full-body MSM obtained using the AMS (AMS. V. 6.0.5.4379; Anybody Technology, Alborg, Denmark) was used for the analysis. The following was the method used for developing the AMS: the thorax was divided into 33 parts, including 12 thoracic vertebrae, 10 pairs of ribs, and the sternum. Trunk muscles, including 15 individual muscles and 328 fascicles and 40 types of lower extremity muscles, were corrected for attachment points and pathways based on magnetic resonance imaging data [25, 29, 30] (Fig 2).

The model was previously validated for the accuracy of the predicted HCF under dynamic conditions using inverse dynamics analysis [25, 29].

### Spinopelvic lower limb sagittal alignment input and simulation process

**Fig 3:**
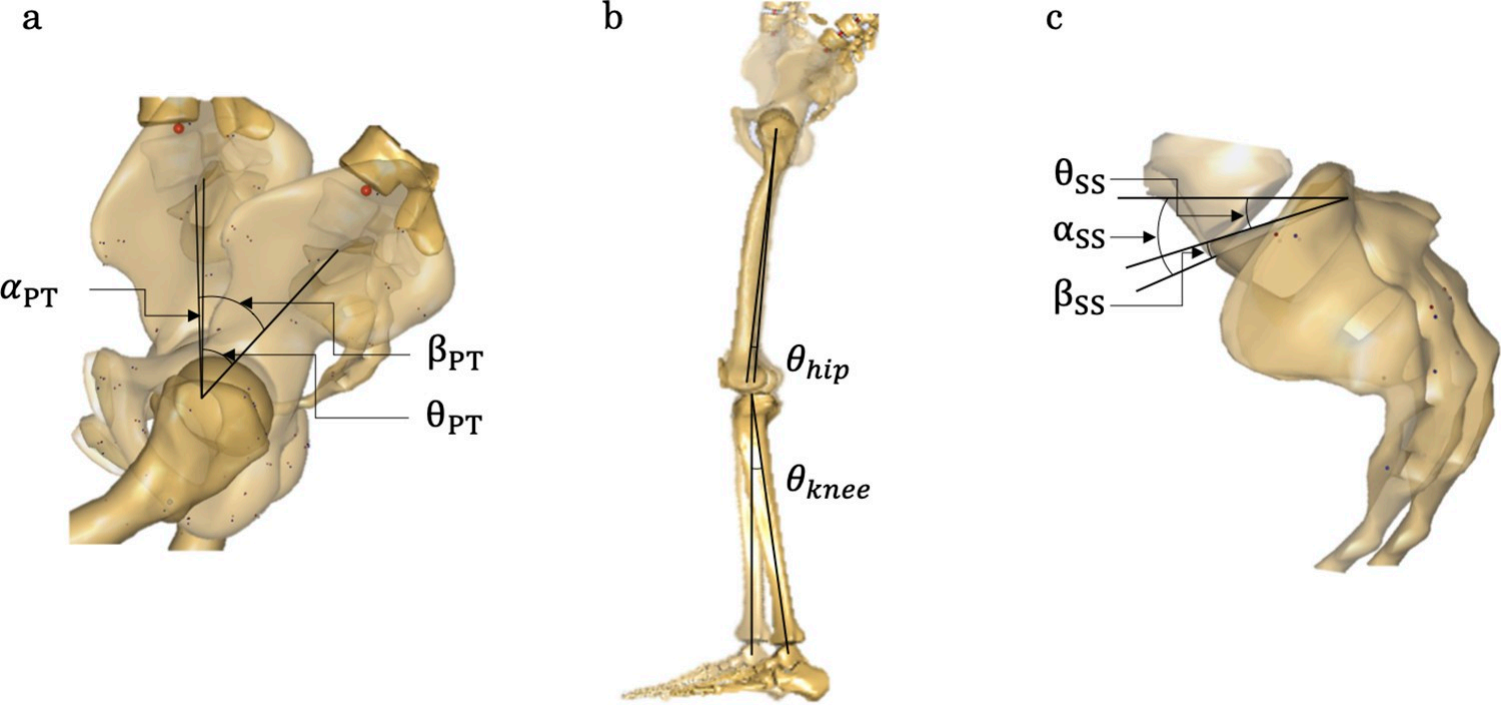
The method for simulating pelvic lower limb alignment in the musculoskeletal models. “θ” is the difference between the model default angle “α” and the patient’s actual measured value “β,” indicating the actual angle to be input into the model. Input “θ_PT_” to be the patient measured value “β_PT_” (a). Input the values of “θ_hip_” “θ_knee_” for hip and knee joint angles (b). Input “θ_ss_” to be the patient measured value “β_SS_” (c).

The input for the sagittal alignment of the spine was based on a previously reported method [31]. The center of the vertebral body was identified as the intersection of the diagonal lines of the quadrilateral formed by each vertebral body in the lateral whole-spine radiographs. Thereafter, the angle between the centers of each vertebra was measured, and the respective values for C7 to S1 in the sagittal plane were inputted into the AMS. The method for inputting the pelvis-lower limb alignment is shown in Fig 3.

**Fig 4:**
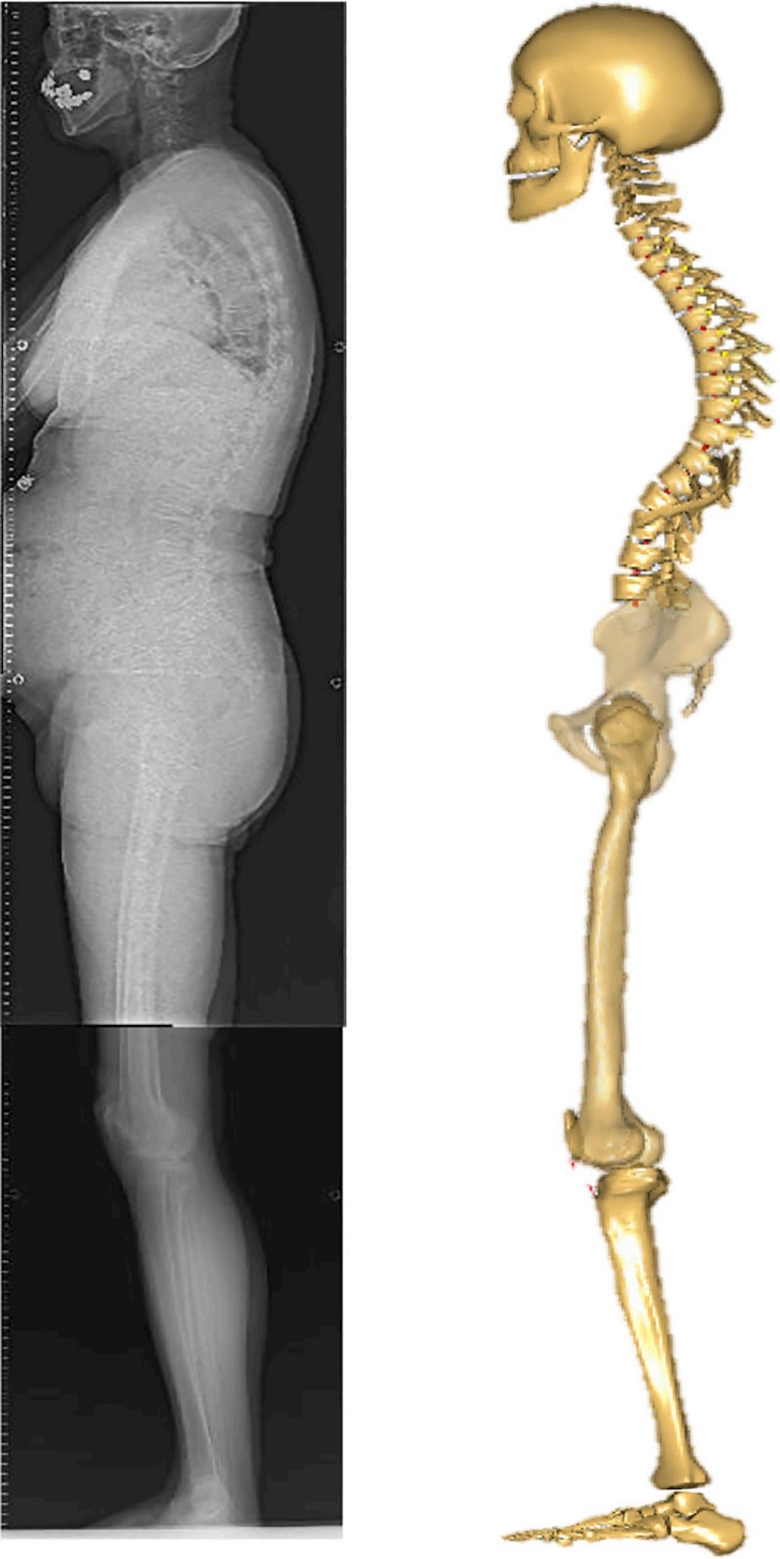
A patient’s full body lateral radiograph and images of the modified musculoskeletal model. This image shows the full-body lateral radiograph of a patient. A modified musculoskeletal model was used to input the patient’s sagittal alignment.

θ is the model input value [deg], α is the value of the default model [deg], and β is the measured value in each subject [deg]. The posture of the subject in the model was corrected based on subject-specific parameters calculated on the radiographs for the PT, SS, FOA, and KFA. The method was applied to all patients, and body weight and height for each patient were inputted into the AMS (Fig 4).

### Model outputs

**Fig 5:**
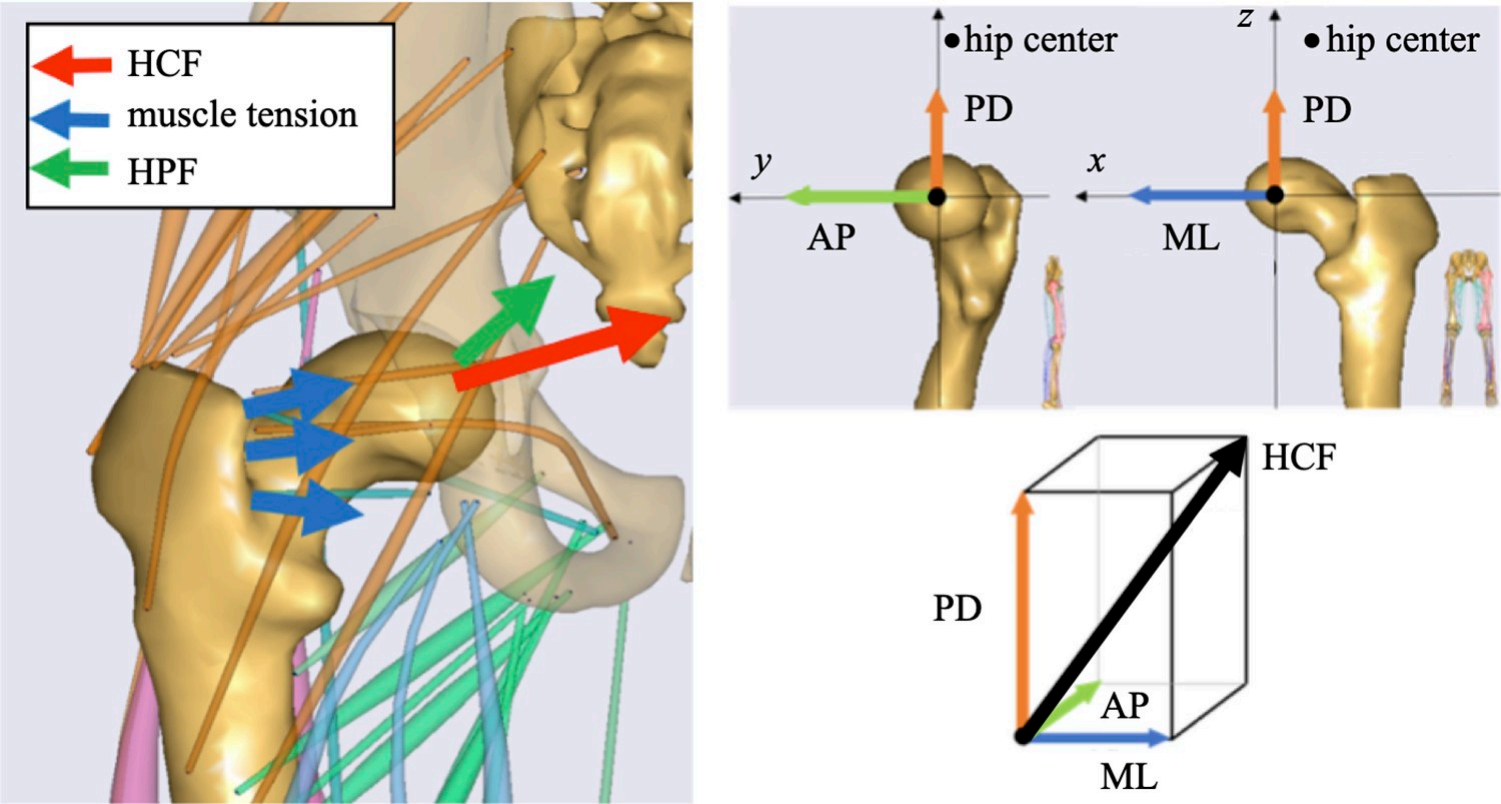
Hip joint diagram for the calculation of muscle contribution to the HCF. The hip penetration force was the force exerted by an adjacent body segment. Muscle tension alone contributed to the HCF. Using the inverse dynamics analysis, the HCF was calculated using three components: anterior/posterior (AP), proximal/distal (PD), and medial/lateral (ML). The HCF was calculated by combining these three force vectors.

Using this model, the HCF, hip flexion, abduction, and external rotation moments in the static standing posture were calculated using inverse dynamics analysis. In the AMS, HCF was defined as shown in Fig 5, and each vector of HCF was calculated using the Eq (1) below.


$$HCF=f+\sum_{i=1}^{n}f_{i},$$ (1)

*f* is the apparent force on the hip joint. *f*_*i*_ is the muscle tension of the muscle attached to the hip joint (Fig 5).

Inertial force, gravity, and ground reaction force are factors that affect the HCF; however, they depend on the model kinematics and mass distribution and are not affected by muscle geometry or forces. Therefore, *f*_*i*_ alone is an individual muscle’s contribution to the HCF [18]. The HCF was calculated by standardization according to each individual’s weight.

### Statistical analysis

All continuous variables are expressed as mean±standard deviations (SDs).

The relationship between the HCF and each parameter was examined using Spearman’s correlation coefficient. Using the International Spine Study Group radiological criteria and Schwab’s realignment objectives for ASD with a cutoff value of 50 mm for the SVA [32], we divided the patients into two groups: the low SVA group with an SVA **≤**50 mm and the high SVA group with an SVA >50 mm. Comparisons between the two groups were performed using the Mann-Whitney U test for continuous variables and the chi-square test for nominal variables. The results were verified using R version 3.5.1 (R Foundation for Statistical Computing, Vienna, Austria). Statistical significance was set at p<0.05.

## Results

Patient characteristics and the calculated HCF and moments are shown in Table 1.

**Table 1:** Clinical characteristics of all patients.

Variable	Value
**Total patients**	20
**Age (years)**	78.3±6.7 (68–92)
**Height (cm)**	147.8±7.8 (134.5–160)
**Weight (kg)**	51.2±10.4 (37.8–82.0)
**BMI (kg/m** ^**2**^ **)**	23.4±4.0 (19.2–32.8)
**TK (°)**	35.0±14.3 (12–67)
**LL (°)**	36.7±17.1 (1–69)
**PT (°)**	26.3±10.6 (12–47)
**SS (°)**	25.8±8.9 (13–38)
**PI (°)**	52.2±9.2 (30–65)
**PI-LL (°)**	15.2±17.9 (-23–44)
**SVA (mm)**	59.7±49.0 (2–162)
**FOA (°)**	5.6±3.3 (0–13)
**KFA (°)**	8.4±5.1 (0.3–12.5)
**HCF (N [%BW])**	168.2±60.1 (70.5–274.4)
**Moment (Nm)**	
**abduction**	1.7±0.2 (1.2–1.8)
**flexion**	1.5±2.8 (-4.7–5.0)
**external rotation**	-0.1±0.3 (-0.7–0.4)

Values are expressed as the mean±standard deviation (range).

BMI, body mass index; TK, thoracic kyphosis; LL, lumbar lordosis; PT, pelvic tilt; SS, sacral slope; PI, pelvic incidence; SVA, sagittal vertical axis; FOA, femur obliquity angle; KFA, knee flexion angle; HCF, hip contact force; N, Newton; BW, body weight.

The mean age of patients was 78.3 years (SD 6.7, range; 68–92), PT 26.3° (SD 10.6, range; 12–47), SVA 59.7 mm (SD 49, range; 2–162), and KFA 8.4° (SD 5.1, range; 0.3–12.5). Compensatory changes were observed, such as pelvic retroversion, anterior trunk shift, and knee flexion. Table 2 shows Spearman’s correlation coefficients between the HCF and spinopelvic lower limb alignment parameters.

**Table 2:** Spearman’s rank correlation coefficients of the HCF with patients’ sagittal alignment parameters.

Variable	Correlation coefficient	p value
**TK (°)**	0.2158	0.3608
**LL (°)**	-0.3281	0.1577
**PT (°)**	-0.0867	0.7161
**SS (°)**	-0.2208	0.3494
**PI (°)**	-0.0726	0.7971
**PI-LL (°)**	0.1857	0.4329
**SVA (mm)**	0.6343	0.0026*
**FOA (°)**	0.4670	0.0387*
**KFA (°)**	0.4945	0.1621

* indicates a significant finding. TK, thoracic kyphosis; LL, lumbar lordosis; PT, pelvic tilt; SS, sacral slope; PI, pelvic incidence; SVA, sagittal vertical axis; FOA, femur obliquity angle; KFA, knee flexion angle.

**Fig 6:**
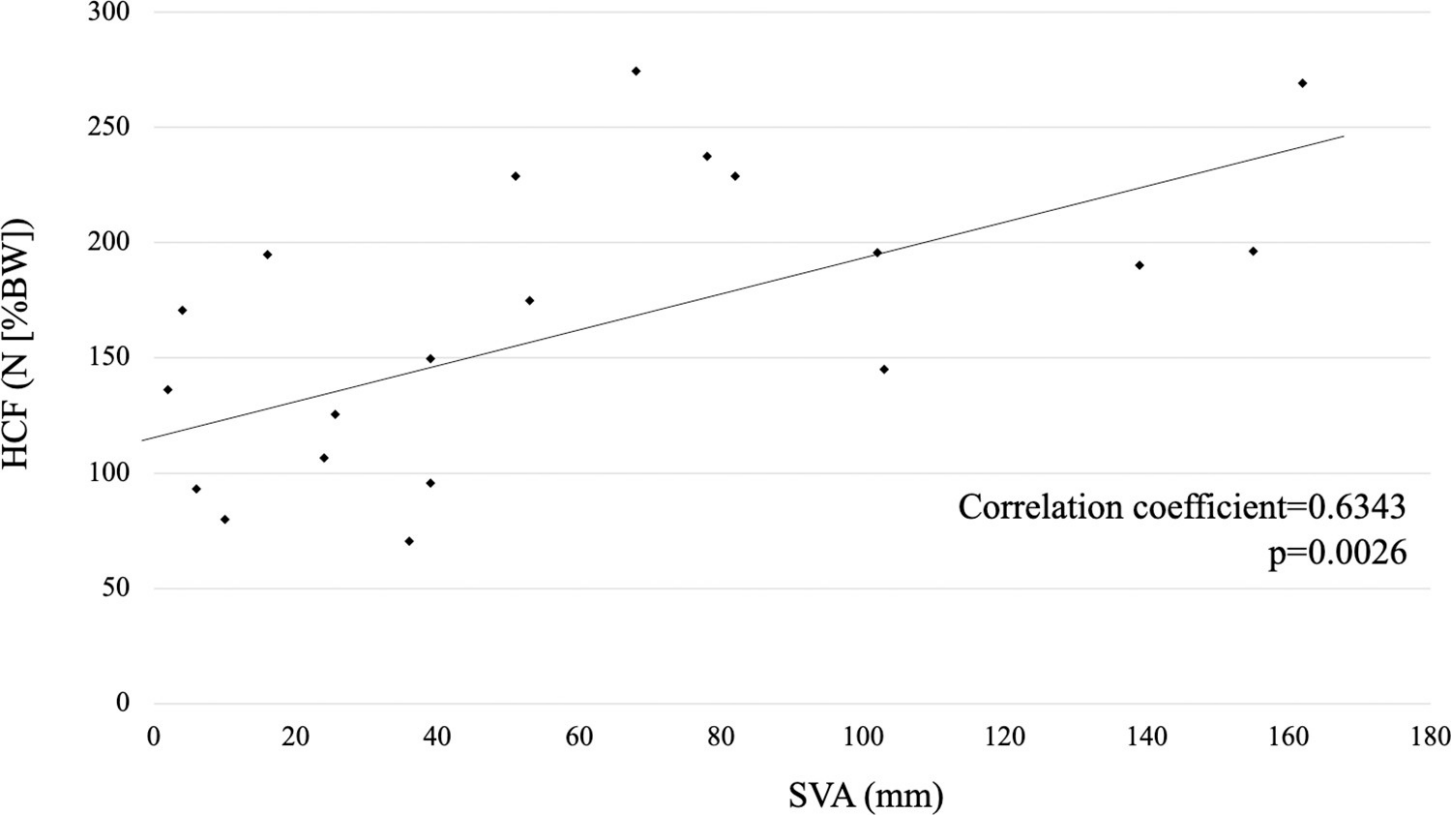
Correlation between the sagittal vertical axis (SVA) and hip contact force (HCF).

The correlation coefficient of SVA was 0.6343 (p = 0.0026), and that of FOA was 0.4670 (p = 0.0387), with the strongest positive correlation in the SVA (Fig 6).

We included 10 patients each in the low SVA and high SVA groups. A comparison of the two groups is shown in Table 3.

**Table 3:** Comparison of the low SVA and high SVA groups.

Variable	Low SVA group	High SVA group	p value
**Total patients**	10	10	
**Age (years)**	74.6±6.1	82.0±5.6	0.0116*
**Height (cm)**	151.5±4.7	144.1±9.2	0.0428*
**Weight (kg)**	56.4±11.3	45.9±7.3	0.0262*
**BMI (kg/m** ^**2**^ **)**	24.5±4.3	22.2±3.7	0.2244
**TK (°)**	34.7±9.80	35.3±18.9	0.9303
**LL (°)**	46.7±15.0	26.7±14.1	0.0067 *
**PT (°)**	25.4±9.1	27.3±13.0	0.7091
**SS (°)**	29.7±7.8	22.0±9.1	0.0578
**PI (°)**	55.1±7.2	49.3±10.9	0.1811
**PI-LL (°)**	8.4±15.5	22.8±19.5	0.0850
**SVA (mm)**	20.1±14.6	99.3±40.6	0.0001*
**FOA (°)**	4.1±3.1	7.1±3.0	0.0435*
**KFA (°)**	6.8±4.7	9.9±5.5	0.1907
**HCF (N [%BW])**	122.2±40.6	214.1±41.1	<0.0001*
**Moment (Nm)**			
**abduction**	1.6±0.2	1.7±0.1	0.5454
**flexion**	0.1±2.7	2.9±2.5	0.0302*
**external rotation**	0.0±0.2	-0.2±0.4	0.3212

Values are expressed as the mean±standard deviation.

* indicates a significant finding.

TK, thoracic kyphosis; LL, lumbar lordosis; PT, pelvic tilt; SS, sacral slope; PI, pelvic incidence; SVA, sagittal vertical axis; FOA, femur obliquity angle; KFA, knee flexion angle; HCF, hip contact force; N, newton; BW, body weight.

The high SVA group showed an older age (p = 0.0116), shorter height (p = 0.0428), lower weight (p = 0.0262), lower LL (p = 0.0067), increased SVA (p = 0.0001), and increased FOA (p = 0.0435) than the low SVA group. HCF values were 122.2 (SD 40.6) (N [%BW]) in the low SVA group and 214.1% (SD 41.1) (N [%BW]) in the high SVA group, with a significant increase of 75.2% in the latter (p<0.0001). No significant differences were noted in abduction and external rotation moments between the groups. However, the flexion moment was significantly increased in the high SVA group compared with that in the low SVA group: 0.1 (SD 2.7) versus 2.9 (SD 2.5) (p = 0.0302).

**Fig 7:**
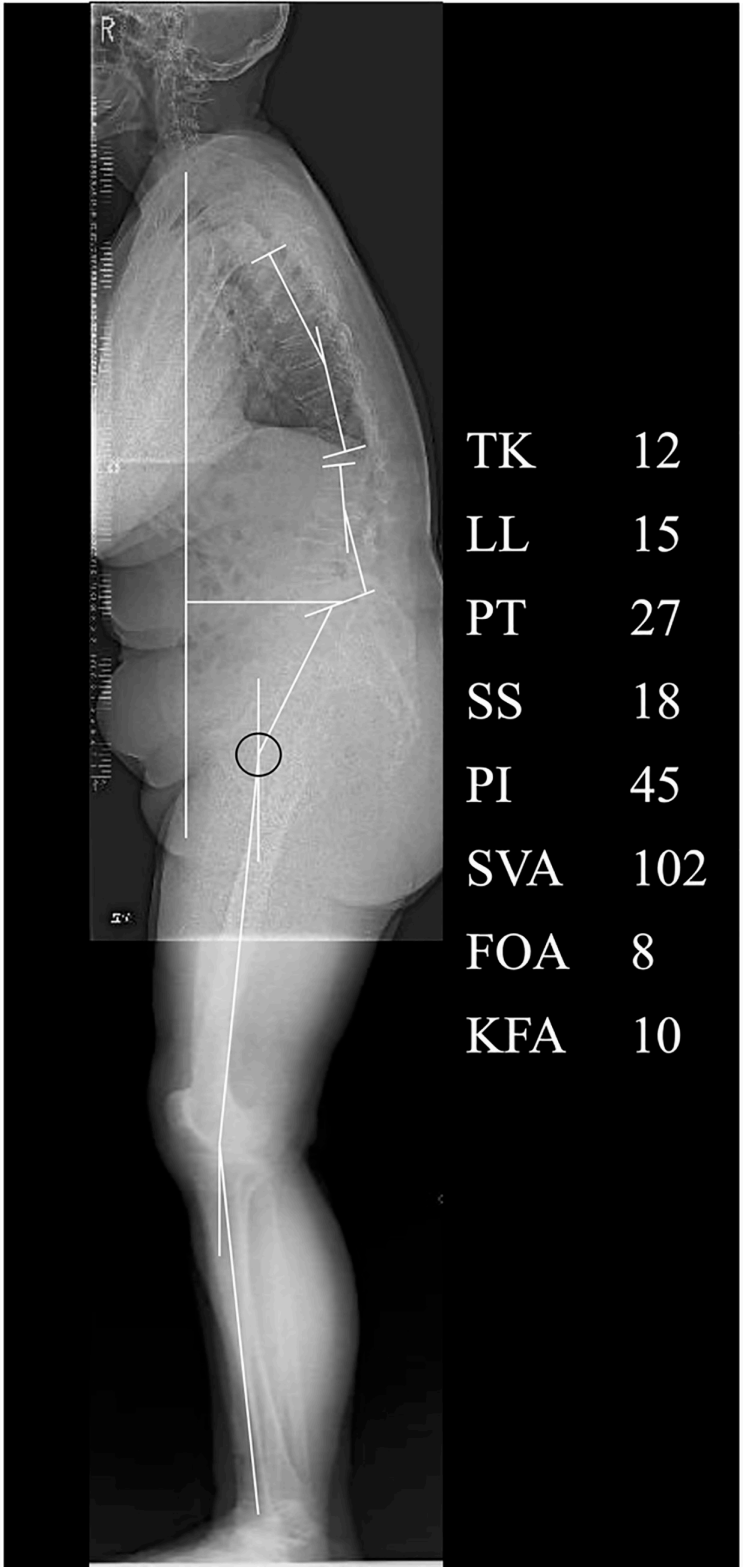
An example case of the high SVA group. Lateral radiographs of the whole spine and lower limb in the standing position.TK, thoracic kyphosis; LL, lumbar lordosis; PT, pelvic tilt; SS, sacral slope; PI, pelvic incidence; SVA, sagittal vertical axis; FOA, femur obliquity angle; KFA, knee flexion angle.

Fig 7 shows a case of high SVA. There was a decrease in lumbar kyphosis, PI-LL mismatch, and an increase in the PT and SVA; compensatory changes in the lower extremities; and a high HCF of 195.7 N [%BW] (Fig 7).

## Discussion

In this study, the relationship between sagittal spinopelvic alignment and the HCF was investigated using a patient-specific adapted MSM of the sagittal spinopelvic lower limb alignment in elderly women’s standing posture. The HCF was positively correlated with the SVA and FOA and was most strongly correlated with the SVA. However, the HCF did not correlate with pelvic alignment. The HCF was increased by 75.2% in patients with an SVA >50 mm compared with the control group. To our knowledge, this is the first study to investigate the relationship between sagittal spinopelvic alignment and the hip joint load.

In this study, we used the AMS, which modified the thorax, changed the muscle attachment points and pathways based on magnetic resonance imaging data, and validated the prediction accuracy of the HCF under dynamic conditions using inverse dynamics analysis [25, 29, 30]. One of our findings was that the HCF in the standing posture was positively correlated with the SVA and FOA. Based on the HCF calculation method in the AMS, the changes in the HCF are considered to be influenced by the tension force that crosses the hip joint and mass distribution [20]. Among them, the muscle activity of the hip associated with postural changes is considered the most significant contributor [33]. In general, in healthy individuals, the gravity line passes posterior or almost center to the hip joint axis [33–36], contributing to reduced muscle activity and hip joint loading in the standing posture [37, 38]. Changes in spinal alignment affect the patient’s sagittal balance and compensate for abnormal pelvic retroversion, extend the hips, and flex the knee to maintain a horizontal sightline [3, 27]. However, in the decompensation phase, wherein compensatory changes are not effective, the patient is sagittally imbalanced as the gravity line moves forward, resulting in an unstable and inefficient standing posture, increased muscle activity, and decreased health-related quality of life [4, 28, 38, 39]. Therefore, the increase in the HCF in patients with a high SVA may be due to an unstable standing posture.

There are no studies on the measurement of the HCF in patients with ASD or biomechanical studies that have investigated the relationship between sagittal spinopelvic alignment and the HCF using an MSM. The HCF was up to twice as high and increased with the progression of hip flexion contracture in MSM analysis [33]. Furthermore, *in vivo* measurement of the HCF with telemeterized hip endoprostheses showed that the HCF was approximately three times higher in the upper body flexion than that in the standing position [40]. In hip flexion contracture and upper body flexion posture, gravity lines can be displaced anteriorly, and the results of this study show that the positive relationship between the SVA and HCF is consistent with the findings of previous studies.

An increase in the hip moment is associated with increased hip joint loading [41]. In the normal standing posture, the hip joint moment was an extension moment because of the gravity line located posterior to the hip axis. Although it has been reported that flexion moments increase in the anterior trunk shift posture because of the anterior shift of the gravity line [41, 42], clinical studies have indicated that a cumulative moment increase in the sagittal plane may be involved in the development of HOA [43]. On the other hand, an increase in the hip abduction moment has also been identified as a risk factor for the development of HOA [43]. Previous studies have reported that the change in hip abduction moment with an increasing hip flexion angle from 0° to 30° was approximately -10–20 Nm and did not significantly change [44]. In addition, it has been shown that the hip external rotation moment changes to internal rotation moment with hip flexion [45]. In our study, the rotational moment was not significantly different between the two groups. However, the internal rotation moment was slightly increased in the ASD group, consistent with the results of the previous study. In this study, only the patient’s sagittal alignment was adapted to the AMS; therefore, the effect of compensatory hip flexion action in the standing position on abduction and rotational moments might not be significant. However, the coronal hip center axis and coronal alignment were not examined. Three-dimensional alignment analysis and individual muscle activity should be considered for detailed moment analysis.

This study has several limitations. First, the results should be interpreted with caution, given the small number of subjects (n = 20). Second, in the AMS, the cervical spine was a single rigid body [24], and the patient’s cervical alignment was not able to adapt to the AMS, which might have influenced the analysis. Third, coronal alignment, individual actual muscle activity, and bone morphology were not considered in the analysis. However, in this study, we focused on showing the relationship between spinopelvic lower limb sagittal alignment and the HCF; therefore, we did not collect electromyography data or perform a comparison with simulated muscle activation in the AMS. Since the AMS has a three-dimensional structure and can be used to analyze muscle activity, future validity studies are needed to adapt three-dimensional patient alignment, bone morphology, and muscle activity to the AMS.

## Conclusions

Our study showed a positive correlation between the HCF and SVA and FOA, and the HCF was most strongly correlated with the SVA but not with pelvic alignment. Our results suggest that changes in sagittal alignment and hip joint loading are closely related and are associated with ASD and worsening HOA. The MSM was found to be a valuable biomechanical tool to noninvasively investigate the relationship between the internal joint load and spinopelvic lower limb parameters. Future studies will need to assess the relationship between hip joint load and sagittal spinopelvic parameters in other poses and dynamic conditions.
